# Sustaining Quality Improvement to Increase Adverse Childhood Experience Questionnaire Response Rates and Proposal of Plan-do-study-act-assess Cycle

**DOI:** 10.1097/pq9.0000000000000920

**Published:** 2026-09-22

**Authors:** Sarah A. Commaroto, Kelly Campbell, Madison Tyle, Melissa Chow, Shainal Gandhi, Monica Khadka, Marilyn Torres, Emily Coughlin, Vinita Kiluk

**Affiliations:** From the * University of South Florida Morsani College of Medicine, Tampa, Fl.; † Department of Pediatrics, University of South Florida, Tampa, Fl.

## INTRODUCTION

Sustainability is a domain of healthcare quality that considers the environmental, social, and financial costs of an intervention.^1^ Sustainability is both an outcome, where the intervention is successfully maintained, and a dynamic process that requires continuous reassessment to ensure the long-term continuation of improved outcomes.^2^ The ultimate goal of a quality improvement (QI) project is for it to become self-sustaining, allowing improvements to persist and patient outcomes to continue to improve even after the intervention is no longer actively implemented. QI projects that provide established methods for designing, implementing, assessing, and embedding changes to services are therefore ideal for developing sustainable healthcare practices.^3,4^ Unfortunately, studies have found that only 45% of QI projects continue delivery of program components, and 33% are not sustained 1 year after completion.^3,5^

Over 2 years, we implemented 2 interventions at 2 resident continuity clinics in the Southeast region to increase ACE Questionnaire (ACE-Q) completion rates.^6^ These interventions are described in Tyle et al.^6^ This report aimed to discuss the sustainability of our QI initiative and assess whether there was a significant change in ACE-Q response rates between the end of our first intervention and the start of our second. We hope to highlight the importance of sustained efforts to achieve desired outcomes in pediatric healthcare settings and propose an extension to the Plan-do-study-act (PDSA) cycle that incorporates a formal reassessment of project goals after implementation. Thus, a PDSA-A (PDSA-assess) could be an improved theoretical framework to ensure that QI projects do not just stop once the intervention is over but instead create lasting change.

## METHODS

Two sequential educational interventions aimed at increasing ACE-Q completion rates, consisting of verbal education for clinic staff and video-based education for caregivers, were implemented over 2 years as part of a single QI initiative, as previously described in Tyle et al.^6^

To evaluate the sustainability of this QI initiative, we conducted a retrospective chart review at two continuity clinics across four discrete time periods corresponding to pre- and postintervention phases for each intervention. Each period included three months of data collection.

For the first cycle, we collected data during a baseline preintervention period (October–December 2021) and a postintervention period (January–March 2022) following implementation of the first educational intervention (staff education). After a period without active intervention, we collected data again, including a second preintervention period (November 2022–January 2023) to assess the durability of the first intervention over time, followed by a second postintervention period (February–April 2023) after implementation of the second educational intervention (a video for caregivers). For each period, we extracted the number of completed ACE-Q forms and the total number of eligible patient visits and calculated completion rates as proportions of eligible patient visits. We then compared completion rates across the four periods, with particular focus on changes between the first postintervention period and the subsequent preintervention period to assess sustainability. Comparisons between time periods were performed using Pearson chi-square tests, with statistical significance defined as *P* < 0.05. Analyses were conducted using SPSS version 29.

## RESULTS

**Fig. 1.**
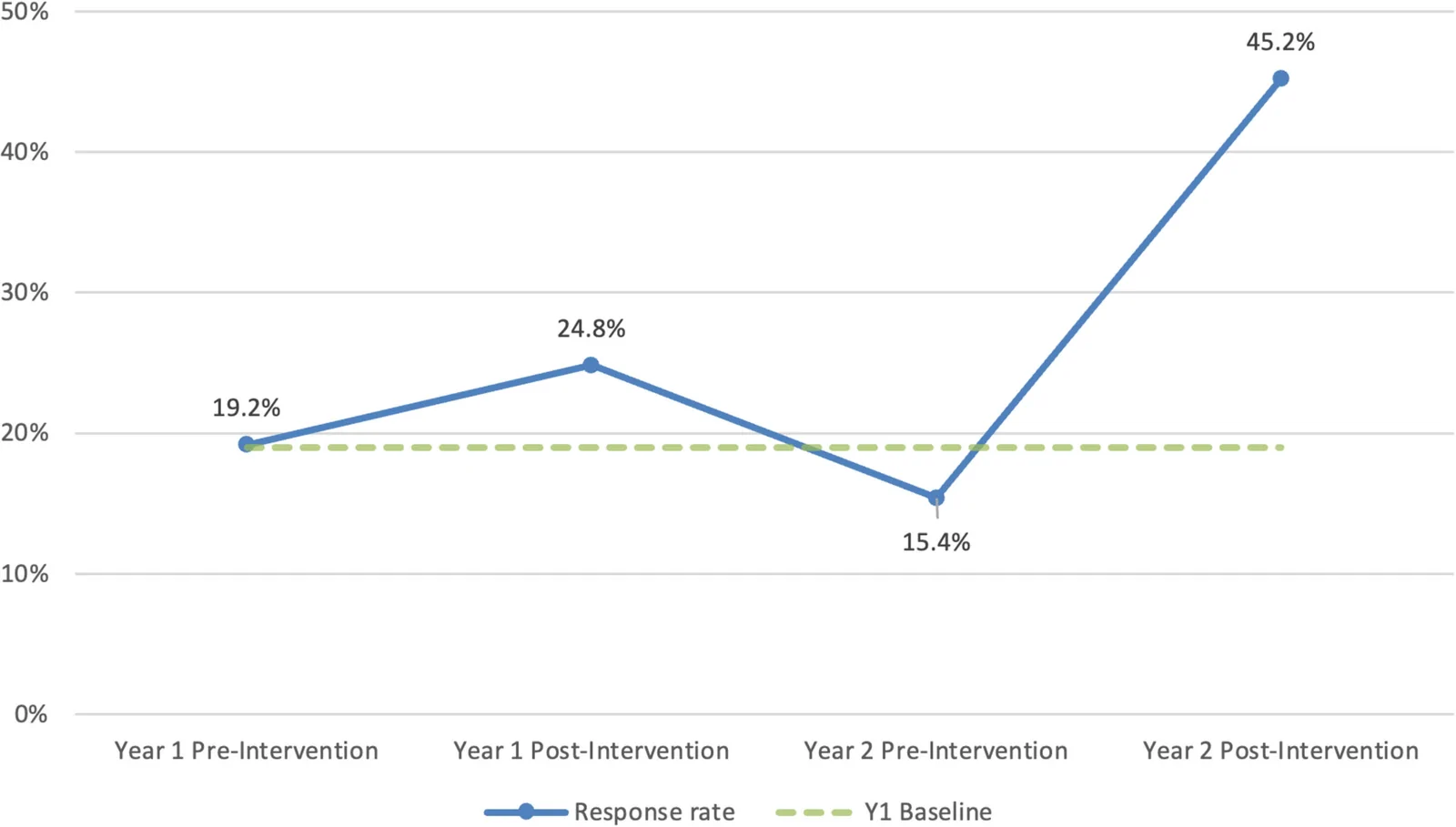
ACE-Q completion rates across study periods. Line graph demonstrating ACE-Q completion rates across 4 study periods: year 1 preintervention, year 1 postintervention (verbal education for staff), year 2 preintervention, and year 2 postintervention (video education for caregivers). The dashed line represents the year 1 baseline completion rate. Completion rates increased after each intervention but declined during the interval between interventions, highlighting challenges in sustaining improvements over time.

**Fig. 2.**
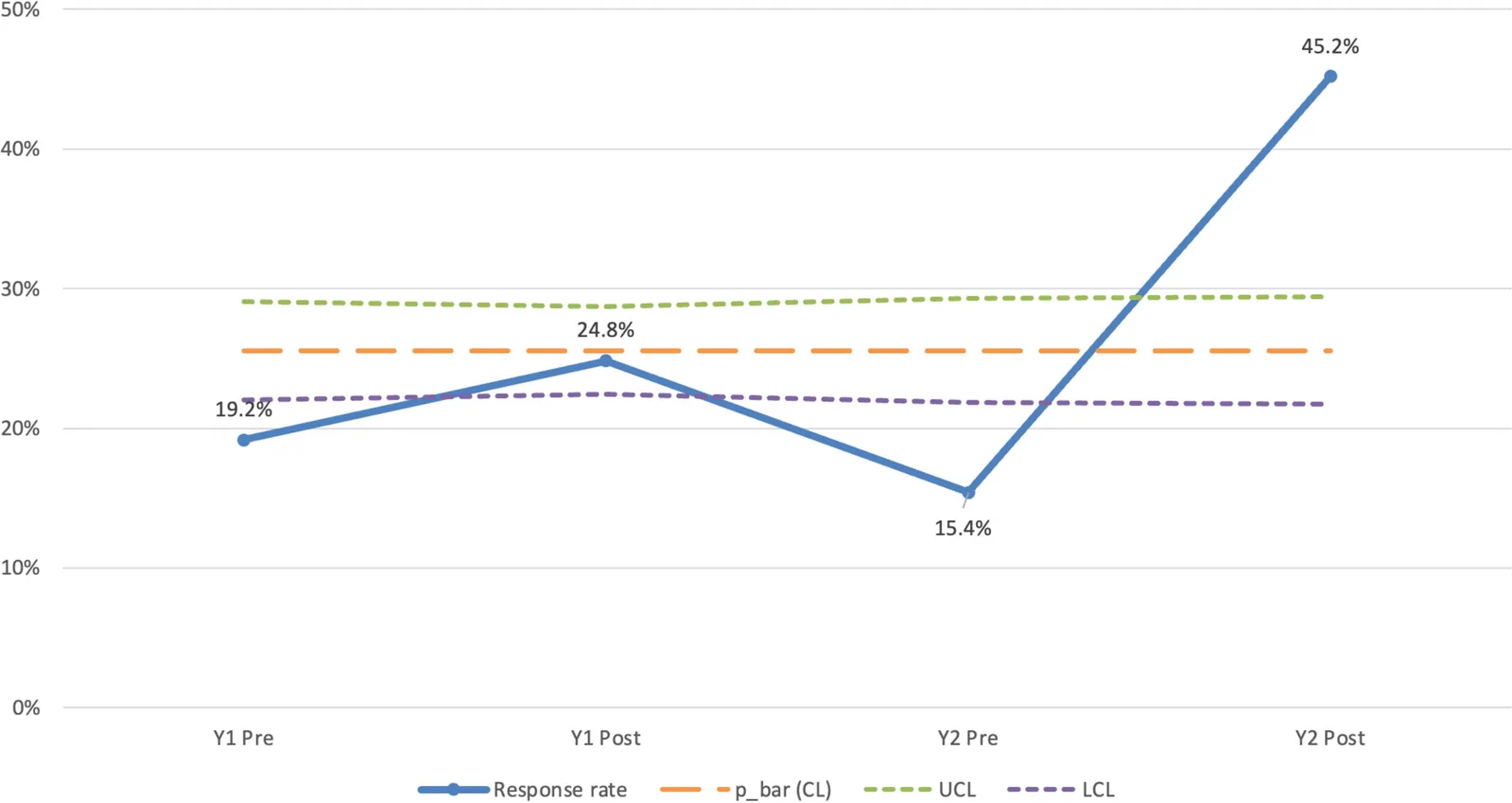
p-chart of ACE-Q completion rates across study periods. p-chart demonstrating variation in ACE-Q completion rates across 4 study periods. Rates increased following each intervention but declined between cycles, indicating limited sustainability. The centerline represents the mean completion rate with corresponding control limits. Weekly variation has been reported previously by Tyle et al.^6^

ACE-Q completion rates increased following each intervention but declined between interventions, indicating variability over time and suggesting limited sustainability of initial improvements (Figs. 1, 2).

## DISCUSSION

Our results highlight dynamic shifts in ACE-Q response rates following our two interventions. Although completion rates improved following each intervention, the decline from Year 1 postintervention to Year 2 preintervention raises concerns about sustainability. Although we used the PDSA method to implement our interventions and achieve the desired change,^7^ our findings suggest that short-term improvements do not necessarily translate into sustained practice change, thus highlighting an important gap in the traditional PDSA framework.

Longitudinal evaluations of pediatric QI initiatives—including early mobility protocols and rapid sequence intubation standardization—demonstrate that sustaining improvements requires ongoing assessment beyond the initial implementation period.^8,9^ These studies incorporated continued monitoring of process and outcome measures, identified emerging barriers, and used follow-up data to refine interventions and guide future educational efforts.^8,9^ Together, these findings support incorporating longitudinal assessment and periodic re-evaluation into the QI process rather than concluding evaluation at the end of the Act stage.

Although multiple PDSA cycles are often required to achieve successful change, neither continued cycling nor the “Act” phase explicitly calls for long-term re-evaluation of a QI intervention’s success. Rather, the Act stage results in 1 of 3 actions: (1) adapt, the desired outcome will be changed and studied further; (2) adopt, the desired outcome will not be studied further and will become a part of normal processes; or (3) abandon, the desired outcome will not be studied further and will not become a part of normal processes.^10^ If a project adopts the change, there is no guarantee it will continue to take hold a month later, let alone years after the immediate adoption period. The PDSA framework does not account for regression, suboptimal adoption, or the potential for a project to be abandoned once initial outcomes are achieved. With sustainability defined as “the maintenance of program activities,”^2^ the PDSA framework does not explicitly incorporate sustainability and lacks a formal mechanism to assess whether maintenance has been achieved.^11^ We therefore propose the PDSA-A cycle to evaluate the continued execution of initiative activities and determine whether sustainability is truly achieved.

The Assess phase should be planned for at the end of the final successful “Act” stage, during which key team members establish predefined intervals to evaluate the project’s long-term sustainability. The initial sustainability assessment should determine whether the program continues to meet its original goals and end-of-cycle performance measures. Ideally, monitoring should continue until performance stabilizes or the intervention is no longer effective, a timeframe that may range from several months to several years after implementation.^11^

Importantly, the proposed Assess phase should extend beyond measuring whether process metrics remain stable over time. Recent qualitative work found that interventions were most likely to be sustained when they became embedded within routine clinical practice and organizational culture, strengthened communication and decision-making, incorporated standardized processes, and received leadership support and stakeholder buy-in.^12^ Conversely, variation in clinical workflows, rapidly changing practice environments, and limited stakeholder engagement were identified as important barriers to long-term success.^12^ Accordingly, in line with these findings and other sustainability frameworks, the Assess phase should evaluate whether the intervention continues to achieve its intended clinical purpose, identify barriers to sustained implementation, engage frontline staff and leadership, and determine whether additional interventions or adaptations are required, possibly leading to another PDSA-A cycle.^13^ In this way, the Assess phase serves as a structured sustainability checkpoint that informs future QI efforts rather than functioning solely as another measurement step. Existing sustainability frameworks can complement the Assess phase by providing guidance for evaluating implementation context and factors that influence long-term maintenance.^2,11^

The PDSA-A cycle adheres to continuous QI methodology, which requires internal follow-up measurement as long as the intervention or program remains useful to the organization.^11^ Long-term follow-up evaluations are scarce in healthcare implementation science and are frequently deferred to the “need for further research” section rather than being systematically incorporated into study design. Several studies have documented challenges in sustaining positive outcomes following improvement initiatives, and reported analyses of sustained effects are rare.^2,5,11^ Our findings align with prior studies demonstrating that discontinuation of interventions, such as checklist use, results in deterioration of achieved improvements and regression to preintervention practices.^14^ Numerous sustainability guidelines have been proposed,^3,5,11,13^ with existing studies identifying and addressing threats to sustainability and, importantly, emphasizing the need to incorporate follow-up analyses into initial study design and to implement ongoing measurement to support sustained success. However, the literature remains highly heterogeneous, and these approaches are not directly integrated into QI project planning because they are not explicitly embedded within the PDSA cycle. No framework currently operates in tandem with the widely used PDSA cycle to explicitly assess sustainability after an intervention has achieved its initial desired outcomes.

Overall, QI projects should always include a sustainability plan. Although sustainability frameworks exist, they are not explicitly integrated into the widely used PDSA framework. Our analysis highlights the need to monitor the effectiveness of a QI project once the intervention ends. Projects that may have been deemed successful by one measure may fail to continue improving patient outcomes in the long term. Although our study was a small, local QI project, the lack of sustainability opens the discussion of how we can implement lasting change within our healthcare system. We herein propose the PDSA-A cycle to include assessment of long-term change to determine whether sustainability is achieved and to monitor outcomes until the desired QI outcome becomes the standard of care.
